# Hereditary Neuropathy with Liability to Pressure Palsy: A Recurrent and Bilateral Foot Drop Case Report

**DOI:** 10.1155/2013/230541

**Published:** 2013-10-23

**Authors:** Filipa Flor-de-Lima, Liliana Macedo, Ricardo Taipa, Manuel Melo-Pires, Maria Lurdes Rodrigues

**Affiliations:** ^1^Department of Pediatrics, Centro Hospitalar do Alto Ave, Hospital de Guimarães, 4835-044 Guimarães, Portugal; ^2^Department of Pediatrics, Centro Hospitalar de São João, Alameda Prof. Hernâni Monteiro, 4200-319 Porto, Portugal; ^3^Unit of Neuropathology, Hospital de Santo António, Centro Hospitalar do Porto, 4099-001 Porto, Portugal; ^4^Department of Neurology, Centro Hospitalar do Alto Ave, Hospital de Guimarães, 4835-044 Guimarães, Portugal

## Abstract

Hereditary neuropathy with liability to pressure palsy is characterized by acute, painless, recurrent mononeuropathies secondary to minor trauma or compression. A 16-year-old boy had the first episode of right foot drop after minor motorcycle accident. Electromyography revealed conduction block and slowing velocity conduction of the right deep peroneal nerve at the fibular head. After motor rehabilitation, he fully recovered. Six months later he had the second episode of foot drop in the opposite site after prolonged squatting position. Electromyography revealed sensorimotor polyneuropathy of left peroneal, sural, posterior tibial, and deep peroneal nerves and also of ulnar, radial, and median nerves of both upper limbs. Histological examination revealed sensory nerve demyelination and focal thickenings of myelin fibers. The diagnosis of hereditary neuropathy with liability to pressure palsy was confirmed by PMP22 deletion of chromosome 17p11.2. He started motor rehabilitation and avoidance of stressing factors with progressive recovery. After one-year followup, he was completely asymptomatic. Recurrent bilateral foot drop history, “sausage-like” swellings of myelin in histological examination, and the results of electromyography led the authors to consider the diagnosis despite negative family history. The authors highlight this rare disease in pediatric population and the importance of high index of clinical suspicion for its diagnosis.

## 1. Introduction

Hereditary neuropathy with liability to pressure palsy (HNPP) is an autosomal dominant disorder characterized by acute, painless, recurrent mononeuropathies that are secondary to minor trauma or compression [1]. In general, typical episodes of palsy begin in the second or the third decades of life. Cases with symptomatic HNPP in the first decade are very rarely reported and frequently underestimated, mainly when there is no family history [1–3]. The prevalence is unknown, mainly due to the inexistence of detailed epidemiological studies, and it has been estimated in 2–16/100000 [3]. The disease is associated with deletions in chromosome 17p11.2, where the peripheral myelin protein 22 (PMP22) gene is localized [1]. The authors report a case of a 16-year-old boy with recurrent and bilateral foot drop.

## 2. Case Report

A 16-year-old Caucasian boy, with no relevant past history except cannabis consumption and no relevant family history, was admitted to the emergency department with paresthesia of the lateral aspect of the right leg and upper surface of the right foot associated with foot drop after minor motorcycle accident. He had steppage gait and limitation of dorsiflexion of right foot. The remaining physical examination was normal. An extensive laboratory testing was performed (white blood cells and platelets count, sedimentation velocity, C-reactive protein, liver function, serum ionogram, phosphorous, calcium, magnesium, muscular enzymes, iron metabolism, folic acid and vitamin B12, thyroid function, immunoglobulins, autoantibodies, celiac disease screening, viral markers, syphilis, and *Borrelia burgdorferi *and *Rickettsia conorii* serologies) with normal results. Lower limb and foot X-ray were normal as well as magnetic resonance of vertebral column. Electromyography revealed conduction block and slowing velocity conduction of right deep peroneal nerve at fibular head (Table 1). He started motor rehabilitation with complete recovery.

**Figure 1:**
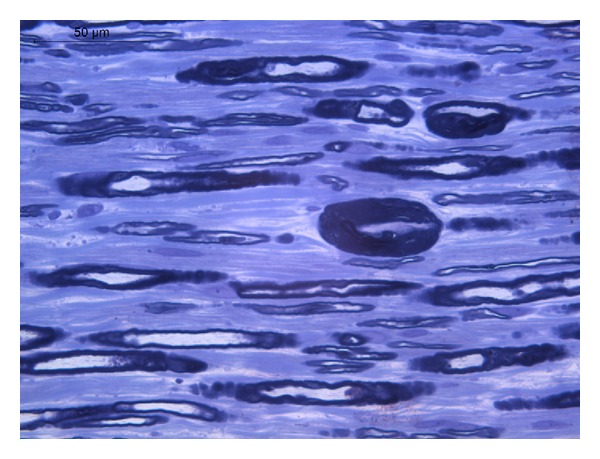
Longitudinal section of sural nerve biopsy showing two myelin outfoldings (tomacula). Toluidine blue stain.

Six months later, he was admitted for the second time to the emergency department with left foot drop and no sensitivity changes after prolonged squatting position. The remaining physical examination was normal; laboratory testing and imaging study were repeated and the results were normal. Electromyography revealed enlarged potential and low amplitude of the left peroneal and sural nerves as well as decreased speed driving of left posterior tibial nerve and increased distal latency, low amplitude, and conduction velocity with enlarged potential of the left deep peroneal nerve (Table 1). Histological examination of sural nerve biopsy revealed predominantly demyelinating changes, having the remaining myelin fibers focal thickenings, the so-called tomacula (Figure 1).

In context of recurrent and bilateral foot drop, he repeated electromyography of the right lower limb that showed the same changes as the left one. An electromyography of ulnar, radial, and median nerves of both upper limbs was performed and showed low amplitude and conduction velocity of sensory and motor components of those nerves (Table 1). The genetic testing confirmed the diagnosis of HNPP showing PMP22 deletion of chromosome 17p11.2. He started motor rehabilitation and avoidance of stressing factors with progressive recovery. After one-year followup, he is completely asymptomatic with normal neurological examination.

## 3. Discussion

Foot drop is a common and distressing problem that can lead to falls and injury and it can be caused by nerve injury, muscle or nerve disorders, or brain and spinal cord disorders [4]. Peripheral neuropathies typically develop with bilateral, symmetric, predominantly distal involvement. They can be inherited or acquired being the last one caused by infections (e.g., Lyme disease); inflammatory diseases (e.g., Guillain-Barré syndrome); rheumatic diseases (e.g., Churg-Strauss syndrome, Henoch-Schönlein purpura, inflammatory bowel disease, juvenile idiopathic arthritis, polyarteritis nodosa, sarcoidosis, and systemic lupus erythematosus); organ failure (e.g., renal or hepatic failure); endocrine abnormalities (e.g., diabetes mellitus and hypothyroidism); disorders of the gastrointestinal tract (e.g., celiac disease); vitamin deficiency or excess or medications (e.g., antibiotics and antiretroviral agents). There were no personal and environmental history related to contact with toxins and no data was found between cannabis consumption and peripheral neuropathy [5]. A meticulous neurological evaluation goes a long way to ascertain the site of the lesion. Nerve conduction and electromyographic studies are useful adjuncts in localizing the site of injury, establishing the degree of damage, and predicting the degree of recovery. Imaging is important in establishing the cause of foot drop be it at the level of the spine, along the course of the sciatic nerve, or in the popliteal fossa [4]. The authors excluded the most common causes of acquired peripheral neuropathy after laboratory and imaging studies.

HNPP is characterized by repeated focal pressure neuropathies such as carpal tunnel syndrome and peroneal palsy with foot drop. PMP22 is the only gene known to be associated with HNPP. A contiguous gene deletion of chromosome 17p11.2 that includes PMP22 is present in approximately 80% of affected individuals; the remaining 20% have a mutation in PMP22. Males and females are equally affected [6]. Approximately one-third of deletion carriers unambiguously detected on the basis of electrophysiological criteria and confirmed by genetic analysis are asymptomatic and do not display significant signs at clinical examination. Thus, the family history is often uninformative, and a significant proportion of probands may be considered as apparently sporadic cases. However, a close questioning and examination of the relatives provided evidence for autosomal dominant inheritance in families that were originally stated by the probands to be normal [7]. Therefore, HNPP can easily be overlooked in those cases in which familial involvement is not recognized unless intensive ascertainment techniques are used. Although HNPP is usually presented as an autosomal dominant trait, sporadic cases carrying a de novo deletion have been described but the percentage of cases of HNPP due to de novo deletion is unknown [7]. In this clinical report, all family members were asymptomatic and genetic testing was not performed. The most common site of focal neuropathy is the peroneal nerve at the fibular head causing foot drop [6]. However, over the years, several atypical clinical presentations of HNPP have been described, suggesting that the diagnosis may not be self-evident, especially when the clinical presentation is without pressure palsies [8]. The electrophysiological pattern of HNPP is characterized by a nonuniform demyelinating polyneuropathy with accentuated distal slowing in some nerves, multifocal conduction slowing at sites of entrapment, and mildly reduced conduction velocities of other segments of motor nerves [1]. In this case report, the characteristic aspect of “sausage-like” swellings of myelin in histological examination after biopsy together with the results of electromyography led the authors to consider the diagnosis of HNPP despite the absence of family history. 

Risk factors for pressure palsies and thus activities that should be avoided include prolonged sitting with legs crossed, occupations requiring repetitive movements of the wrist, prolonged leaning on elbows, and rapid weight loss. Prevention of primary manifestations includes protective pads at elbows or knees to prevent pressure and trauma to local nerves, and ankle-foot orthoses may alleviate foot drop. Full recovery over a period of days to months occurs in approximately 50% of episodes. Incomplete recovery is fairly common, but the remaining symptoms are rarely severe. Poor recovery correlates with a history of prolonged focal compression of the nerve [6]. Although no pharmacological treatment has been known to be beneficial, Hock Heng et al. described rapid and almost complete recovery from the longstanding weakness after corticosteroid therapy in two pediatric patients [9].

During the first attack, diagnosis is often overlooked and delayed, especially when the family history is not available or negative and when detailed electrophysiological examinations are not performed. In our case report, the first episode of foot drop was associated with the motorcycle accident. In fact, the right deep peroneal nerve at the fibular head on the electromyography was not normal, but this result was compatible to traumatic event. After the second foot drop episode, at this time in the opposite site of lower limb and after prolonged squatting, the authors thought about this disorder together with the biopsy results. Data from electromyography of upper and lower limbs made the diagnosis more consistent, which was confirmed by genetic testing. The authors highlight this rare disease in pediatric population and the importance of high index of clinical suspicion for its diagnosis.

## Figures and Tables

**Table 1a:** (a)

Motor nerve conduction study			
Nerve	CMAP amplitude (SD) mV	CV (SD) m/s	L1 (SD) ms
First episode			
Tibial			
Ref.	5.8 (1.9)	43.6 (5.1)	3.96 (1)
Right	Ankle-AHM 6.8 Knee-ankle 3.1	Knee-ankle 40.0	Ankle-AHM 4.5 Knee-ankle 14.5
Peroneal			
Ref.	5.1 (2.3)	39.4 (5.8)	5.5 (1.2)
Right	Ankle-EDB 5.3 Ankle 3.0 Below knee 1.6	Ankle 41.1 Below knee 29.4	Ankle-EDB 11.8 Ankle 13.5 Below knee 14.5

Second episode			
Tibial			
Ref.	5.8 (1.9)	43.6 (5.1)	3.96 (1)
Right	Ankle-AHM 8.4 Knee-ankle 8.0	Knee-ankle 41.8	Ankle-AHM 5.2 Knee-ankle 14.3
Left	Ankle-AHM 11.8 Knee-ankle 7.7	Knee-ankle 36.9	Ankle-AHM 3.5 Knee-ankle 13.8
Peroneal			
Ref.	5.1 (2.3)	39.4 (5.8)	5.5 (1.2)
Right	Ankle-EDB 3.8 Ankle 4.3 Below knee 4.0	Ankle 43.1 Below knee 33.3	Ankle-EDB 6.0 Ankle 12.5 Below knee 14.0
Left	Ankle-EDB 5.1 Ankle 4.0 Below knee 3.6	Ankle 42.5 Below knee 36.4	Ankle-EDB 4.9 Ankle 12.2 Below knee 13.3
Median			
Ref.	7 (3)	55.6 (2.9)	3.49 (0.34)
Right	Wrist-APB 5.0 Below elbow-wrist 4.9	Below elbow-wrist 41.5	Wrist-APB 4.6 Below elbow-wrist 8.7
Left	Wrist-APB 4.7 Below elbow-wrist 4.3	Below elbow-wrist 43.4	Wrist-APB 4.2 Below elbow-wrist 8.3
Ulnar			
Ref.	5.5 (2)	57.8 (2.1)	6.1 (0.69)
Right	Wrist-ADM 5.2 Above elbow-below elbow 4.3 Plexus-above elbow 4.2	Below elbow-wrist 45.0 Above elbow-below elbow 32 Plexus-above elbow 42.9	Wrist-ADM 2.7 Above elbow-below elbow 9.2 Plexus-above elbow 9.9
Left	Wrist-ADM 5.8 Above elbow-below elbow 4.4 Plexus-above elbow 4.2	Below elbow-wrist 54.5 Above elbow-below elbow 41.7 Plexus-above elbow 45.8	Wrist-ADM 3.2 Above elbow-below elbow 7.7 Plexus-above elbow 8.9
Radial			
Ref.	8.79 (2.3)	66.2 (7.3)	2.46 (0.72)
Right	Below elbow 10.5 Above elbow-below elbow 7.6	Below elbow 58.7	Below elbow 1.79 Above elbow-below elbow 5.2
Left	Below elbow 10.2 Above elbow-below elbow 7.8	Above elbow-below elbow 57.0	Below elbow 1.7 Above elbow-below elbow 5.1

**Table 1b:** (b)

Sensory nerve conduction study			
Nerve	Amplitude (SD) uV	L1 (SD) ms	CV (SD) m/s
First episode			
Sural			
Ref.	18.67 (4.39)	2.79 (0.45)	31.8 (5.6)
Right	4.7	4.3	45.2

Second episode			
Sural			
Ref.	18.67 (4.39)	2.79 (0.45)	31.8 (5.6)
Right	Ankle-foreleg 2.5	Ankle-foreleg 3.9	Ankle-foreleg 44.0
Left	Ankle-foreleg 2.6	Ankle-foreleg 4.0	Ankle-foreleg 44.0
Median			
Ref.	38.4 (15.6)	2.84 (0.34)	65.8 (3.8)
Right	Digiti II-wrist 9.6	Digiti II-wrist 3.8	Digiti II-wrist 46.7
Left	Digiti II-wrist 25	Digiti II-wrist 4.0	Digiti II-wrist 43.8
Ulnar			
Ref.	35.4 (14.7)	5.67 (0.59)	67.1 (4.7)
Right	Digiti V-wrist 13	Digiti V-wrist 3.3	Digiti V-wrist 48.0
Left	Digiti V-wrist 20	Digiti V-wrist 3.3	Digiti V-wrist 46.2
Radial			
Ref.	21.4 (4.8)	2.6 (0.3)	58.1 (4.7)
Right	Interosseous digiti-forearm 22	Interosseous digiti-forearm 2.8	Interosseous digiti-forearm 44.5
Left	Interosseous digiti-forearm 20	Interosseous digiti-forearm 3.0	Interosseous digiti-forearm 42.3

CMAP: compound motor action potential; CV: conduction velocity; L1: onset latency; SD: standard deviation; AHM: abductor hallucis muscle; EDB: extensor digitorum brevis; APB: abductor pollicis brevis; ADM: abductor digiti minimi.
